# Supplementary material for: TSFF-Net: A deep fake video detection model based on two-stream feature domain fusion
Source: PLoS One. 2024 Dec 13;19(12):e0311366. doi: 10.1371/journal.pone.0311366 (PMC11642989; doi:10.1371/journal.pone.0311366)
Supplement: Attachment — Submitted filename: Response to Reviewers.docx [file pone.0311366.s001.docx]

**Original Manuscript ID: PONE-D-24-22578**

**Original Article Title: “TSFF-Net: A Deep Fake Video Detection Model Based on Two-Stream Feature Domain Fusion”**

**To:** Editor

**Re:** Response to reviewers

Dear Editor,

Thanks for your letter and review work on our manuscript entitled “TSFF-Net: A Deep Fake Video Detection Model Based on Two-Stream Feature Domain Fusion”. We greatly appreciate the reviewers’ complimentary comments and suggestions. These comments are very helpful and valuable for improving and polishing our paper. The point-by-point responses to reviewers’ comments are given in the following part.

We have added additional information about the datasets in the Methods section and included a statement confirming that our data collection and analysis methods comply with the terms and conditions set by the data sources.

We confirm that our submission contains all the raw data required to replicate the results of the study, in accordance with PLOS's definition of the minimal data set. Specifically, the code used in this study is publicly available at: <https://github.com/hwZHc/TSFF-Net.git>, and all relevant data, metadata, and methods are provided within the manuscript and its Supporting Information files. This ensures that all study findings can be fully replicated.

Best regards,

Hangchuan Zhang, Caiping Hu, Shiyu Min, Hui Sui and Guola Zhou

**Reviews and Responses**

**Reviewer #1:**

**Point 1:** Many of the citations had problems showing up as 'Error! Reference source not found', such as the citations in the INTRODUCTION section for the datasets FaceForensics++ and Celeb-DF.

**Response 1:** In response to the "Error! Reference source not found" issue you mentioned, we have conducted a thorough examination and correction. We found that the problem occurred in the citation formatting of the reference literature, especially regarding the FaceForensics++ and Celeb-DF datasets mentioned in the "Introduction" section. We failed to format these citations correctly, which resulted in the citations not being displayed properly in the final document. Therefore, we rechecked the format of all citations to ensure they comply with the journal's citation standards. Particularly for the FaceForensics++ and Celeb-DF datasets, we have ensured the correctness of the citation format.

**Point 2:** The proposed method uses efficient-B0 as backbone, has it tried to use other cnn structures such as ResNet or DenseNet?

**Response 2:** Thank you for your insightful comment. In this study, we chose EfficientNet-B0 as the backbone network primarily because it offers a good balance between performance and efficiency. However, we did consider using other CNN architectures, such as ResNet and DenseNet. In preliminary experiments, we tested ResNet and DenseNet but found that their computational complexity was higher, leading to significantly increased training times. Although they could potentially provide slightly higher accuracy in certain cases, we believe that EfficientNet-B0 offers a better trade-off between accuracy and efficiency.

That being said, this does not mean that ResNet or DenseNet cannot be used for this task. In future research, we may further explore these or other more complex network architectures to enhance the model's performance.

**Point 3:** The goal of the proposed method is to detect the latest emergence of Gan-based face forgery, but the datasets FaceForensics++ and Celeb-DF both seem to be based on encoder-decoder structures, not traditional GAN. hopefully the article has a discussion on gan-generated deepfake, for example, [1][2][3].

[1] Synthesizing black-box anti-forensics deepfakes with high visual quality
[2] Adversarial attack on fake-faces detectors under white and black box scenarios
[3] Securing facial bioinformation by eliminating adversarial perturbations

**Response 3:** We appreciate your concern that while our datasets, FaceForensics++ and Celeb-DF, are based on encoder-decoder architectures, they do not represent the traditional GAN structures. We have chosen these datasets because they provide a diverse and high-quality set of deepfake samples, which is essential for assessing the performance of detection algorithms. Regarding the detection of GAN-generated deepfakes, we have included a discussion on this topic in Related works of our manuscript.

**Reviewer #2:**

**Point 1:** The introduction section should further compare the recent research work, especially the research based on transformer and hybrid models, such as RTSMFFDE-HKRR: A fault diagnosis method for train bearing in noise environment.

**Response 1:** We appreciate your suggestion to compare recent research work, we have discussed these models in detail in the Related Work section, we will revise the Introduction section to briefly introduce these models and provide a concise comparison with our research.

**Point 2:** Identify and list research gaps in the related work section. Discuss how the proposed approach fills these gaps and offers advantages.

**Response 2:** We have added relevant descriptions in section 2.2. At the end of Section 2.2, we elaborate on the shortcomings of existing research, which tends to focus more on capturing local features. However, this often overlooks the differences between genuine and forged areas in forged videos. Therefore, we combine facial image features in both spatial and frequency domains, using an improved Vision Transformer (ViT) and cross-attention mechanism for global information modeling, to achieve deepfake detection and enhance the model's performance in scenarios with low quality and high compression rates.

**Point 3:** The model structure diagram in Figure 1 can further refine the specific structure of each branch.

**Response 3:** Thank you for your suggestion. We acknowledge that the model structure diagram (Figure 1) could provide clearer information by further refining the specific structure of each branch. In the revised version, we will update Figure 1 to illustrate the detailed structure of each branch. This will help to better visualize the proposed model architecture and the role of each branch.

**Point 4:** Include a more detailed discussion of model training information, such as loss functions, hyperparameter optimization procedures, etc.

**Response 4:** We have added relevant descriptions in section 4.2.

**Point 5:** Provide details of the image dataset and training parameters in tabular form.

**Response 5:** Thank you for your question. Below is a table that provides details of the image dataset used and the training parameters:

Table 1. Basic information of the dataset used.

| Dataset | Real(video) | Fake (video) | Real(Frame) | Fake(Frame) |
| --- | --- | --- | --- | --- |
| FaceForensics++ | 1,000 | 4,000 | 509.9k | 2,039.6k |
| Celeb-DF | 590 | 5,639 | 225.4k | 2,116.8k |

Table 2. Details of the training parameters.

| Training Parameters | Settings |
| --- | --- |
| Optimizer | SGD |
| Image Resolution | 256x256 pixels |
| Learning Rate | 0.01 |
| Weight Decay | 0.000001 |
| Batch Size | 32 |
